# Functional characterization of TRICHOMELESS2, a new single-repeat R3 MYB transcription factor in the regulation of trichome patterning in *Arabidopsis*

**DOI:** 10.1186/1471-2229-11-176

**Published:** 2011-12-15

**Authors:** Lijun Gan, Kai Xia, Jin-Gui Chen, Shucai Wang

**Affiliations:** 1 College of Life Sciences, Nanjing Agricultural University, Nanjing 210095, China; 2 Key Laboratory of Molecular Epigenetics of MOE and Institute of Genetics & Cytology, Northeast Normal University, Changchun 130024, China; 3 Biosciences Division, Oak Ridge National Laboratory, Oak Ridge, TN 37831, USA; 4 Department of Botany, University of British Columbia, Vancouver, BC V6T 1Z4, Canada

## Abstract

**Background:**

Single-repeat R3 MYB transcription factors (single-repeat MYBs) play important roles in controlling trichome patterning in *Arabidopsis*. It was proposed that single-repeat MYBs negatively regulate trichome formation by competing with GLABRA1 (GL1) for binding GLABRA3/ENHANCER OF GLABRA3 (GL3/EGL3), thus inhibiting the formation of activator complex TTG1(TRANSPARENT TESTA GLABRA1)-GL3/EGL3-GL1 that is required for the activation of *GLABRA2 *(*GL2*), whose product is a positive regulator of trichome formation. Previously we identified a novel single-repeat MYB transcription factor, TRICHOMELESS1 (TCL1), which negatively regulates trichome formation on the inflorescence stems and pedicels by directly suppressing the expression of *GL1*.

**Results:**

We analyzed here the role of TRICHOMELESS2 (TCL2), a previously-uncharacterized single-repeat MYB transcription factor in trichome patterning in *Arabidopsis*. We showed that TCL2 is closely related to TCL1, and like TCL1 and other single-repeat MYBs, TCL2 interacts with GL3. Overexpression of *TCL2 *conferred glabrous phenotype while knockdown of *TCL2 *via RNAi induced ectopic trichome formation on the inflorescence stems and pedicels, a phenotype that was previously observed in *tcl1 *mutants. These results suggested that TCL2 may have overlapping function with TCL1 in controlling trichome formation on inflorescences. On the other hand, although the transcription of *TCL2*, like *TCL1, *is not controlled by the activator complex formed by GL1 and GL3, and TCL2 and TCL1 proteins are more than 80% identical at the amino acid level, the expression of *TCL2 *under the control of *TCL1 *promoter only partially recovered the mutant phenotype of *tcl1*, implying that TCL2 and TCL1 are not fully functional equivalent.

**Conclusions:**

TCL2 function redundantly with TCL1 in controlling trichome formation on inflorescences, but they are not fully functional equivalent. Transcription of *TCL2 *is not controlled by activator complex formed by GL1 and GL3, but *MIR156 *controlled SQUAMOSA PROMOTER BINDING PROTEIN LIKE (SPL) transcription factors. However, SPLs might require co-activators to regulate the expression of their target genes, including *TCL1*, *TRY *and possibly, *TCL2*.

## Background

Trichome patterning in *Arabidopsis *is controlled by several different types of transcription factors. Based on available evidence, it was proposed that an R2R3 MYB-type transcription factor GLABRA1 (GL1) [1], a bHLH transcription factors GLABRA3 (GL3) [2] or ENHANCER OF GLABRA3 (EGL3) [3], and a WD40-repeat protein TRANSPARENT TESTA GLABRA1 (TTG1) [4,5], form an activator complex TTG1-GL3/EGL3-GL1. This activator complex activates the expression of *GLABRA2 *(*GL2*), which encodes a homeodomain protein that promotes trichome formation in shoots [6-8]. The same activator complex also induces the expression of some single-repeat R3 MYB genes.

So far a total of six genes in the *Arabidopsis *genome have been reported encoding single-repeat R3 MYB transcription factors, including *TRIPTYCHON *(*TRY*) [9,10], *CAPRICE *(*CPC*) [11,12], *TRICHOMELESS1(TCL1) *[13], and *ENHANCER OF TRY AND CPC1, 2 *and *3 *(*ETC1*, *ETC2 *and *ETC3/CPL3*) [14-18]. Single-repeat MYB transcription factors are characterized by their short sequence (75-112 amino acids) and consist largely of the single MYB domain [19]. Single-repeat R3 MYBs can move from a trichome precursor cell to its neighboring cells to block the formation of the activator complex by competing with GL1 in binding GL3 or EGL3, thus inhibiting trichome formation in shoots [8,13,18,19]. These single-repeat R3 MYB transcription factors differ in their binding strength to GL3 and their capacity to compete with GL1 for binding GL3. A yeast three-hybrid assays suggested that CPC is the most potent inhibitor followed by ETC1, TRY, ETC3 and ETC2 [18]. A protoplast transfection assay showed that TCL1 is stronger than CPC in their ability to bind GL3 [19].

Although over expressing any of these six single-repeat R3 transcription factors in *Arabidopsis *causes a glabrous phenotype, single mutants of these genes have different phenotypes. Mutation in *TRY *results in trichome cluster phenotype [9,10], mutation in *CPC *increases trichome number [11,12], and mutation in *TCL1 *causes ectopic trichome formation on inflorescence stems and pedicels [13]. Mutation in *ETC1*, *ETC2 *or *ETC3 *does not dramatically affect trichome formation. However, analysis of double, triple and higher order mutants between these single mutants indicated that all six single-repeat MYB transcription factors function in a highly redundant manner to control trichome formation in *Arabidopsis *[13-18,20].

In addition to competing with GL1 for binding GL3, our previous results showed that TCL1 could also directly suppress the expression of *GL1 *[13]. Interestingly, unlike many other single-repeat MYBs, the expression of *TCL1 *is not controlled by the activator complex formed by GL1 and GL3/EGL3. Recently, it has been found that *microRNA156 *(*MIR156*)-targeted SQUAMOSA PROMOTER BINDING PROTEIN LIKE (SPL) 9 can activate *TCL1 *and *TRY *expression through binding to their promoters [21].

Here we analyzed the role of TRICHOMELESS2 (TCL2), a previously uncharacterized single-repeat MYB transcription factor in trichome patterning in *Arabidopsis*. We demonstrated that TCL2, like other single-repeat MYBs, negatively regulates trichome formation. Overexpression of *TCL2 *conferred glabrous phenotype while knockdown of *TCL2 *via RNAi induced ectopic trichome formation on inflorescence stems and pedicels, a phenotype that was previously observed in *tcl1 *mutants. Furthermore we provide genetic evidence that TCL2 and TCL1 may not be fully functional equivalent. We also showed that *MIR156 *is involved in the regulation of *TCL2*, and SPLs may require co-activators to regulate the expression of their target genes, including *TCL1*, *TRY*, and possibly, *TCL2*.

## Results

### TRICHOMELESS2 is closely related to TCL1

**Figure 1:**
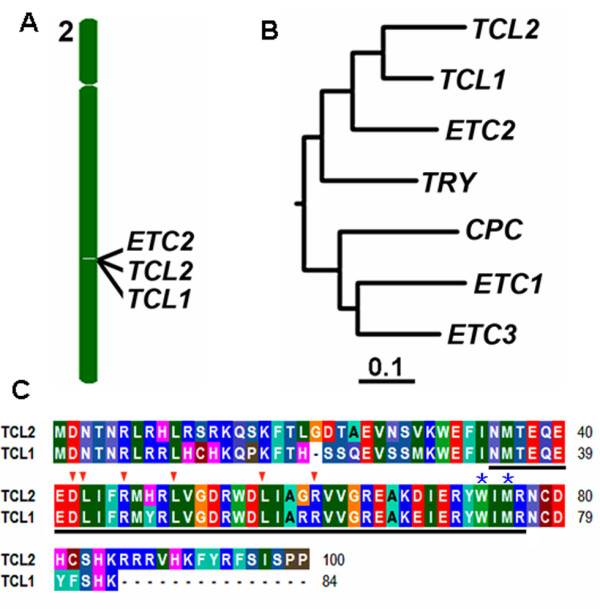
**TCL2 is closely related to TCL1**. **A**. *TCL2 *is a tandem repeat gene with *TCL1 *and *ETC2 *located on chromosome II. **B**. Phylogenetic analysis of the seven single-repeat R3 MYB transcription factors in *Arabidopsis*. The phylogenetic tree using the entire amino acid sequence was generated using software CLUSTAL W with default settings (http://www.genome.jp/tools-bin/clustalw) **C**. Sequence alignment of TCL2 and TCL1. Identical amino acids are shaded in same color. Underlines indicate the R3 MYB domain, arrowheads indicate the amino acid signature [D/E]L × 2[R/K] × 3 L × 6 L × 3R that is required for interacting with R/B-like BHLH transcription factors, and asterisks indicate the amino acids within the MYB domain that are crucial for cell-to-cell movement of CPC.

In *Arabidopsis*, a total of six single-repeat MYB transcription factors have been characterized so far [13]. In order to examine whether there is any uncharacterized single-repeat MYB transcription factors, the entire amino acid sequence of TCL1 was used as a template to BLAST *Arabidopsis *protein database (http://www.ncbi.nlm.nih.gov). In addition to *TRY*, *CPC*, *TCL1*, *ETC1*, *ETC2 *and *ETC3/CPL3 *that have been reported previously, our analysis revealed that this is another single-repeat MYB transcription factor encoded by *At2g30424 *in the Arabidopsis genome. The gene encoding this uncharacterized single-repeat MYB was designated *TRICHOMELESS2 *(*TCL2*). *TCL2 *is nearby two other single-repeat MYB genes, *ETC2 *and *TCL1*, on chromosome II (Figure 1A), with *ETC2 *is a tandem repeat gene with *TCL2*, while *TCL2 *and *TCL1 *is separated by another gene *At2g30430*. Phylogenic analysis using full length protein sequences showed that TCL2 is closely related to TCL1 (Figure 1B). TCL2 shares 80% identify with TCL1 at the amino acid level. As shown in Figure 1C, like TCL1 and other single-repeat MYBs, TCL2 also contains the conserved amino acid signature [D/E]L × 2 [R/K] × 3 L × 6 L × 3R that is required for the interaction with R/B-like bHLH transcription factors[22] and the conserved amino acids that have been shown to be required for the cell-to-cell movement of the single-repeat MYB transcription factor CPC [23].

### Expression and subcellular localization of TCL2

**Figure 2:**
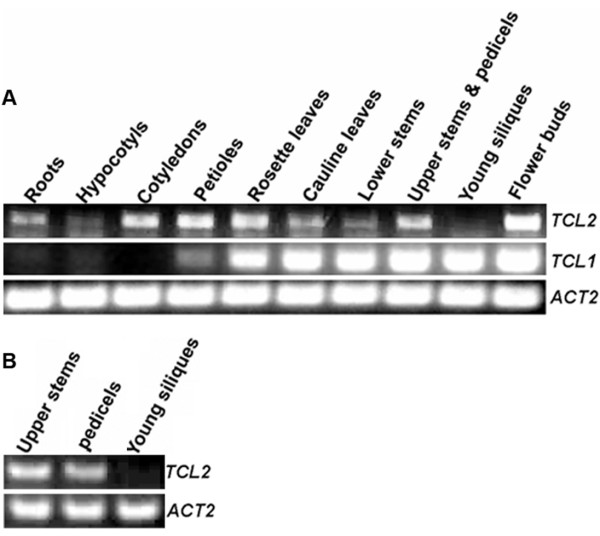
**Expression of *TCL2 *and *TCL1 *in *Arabidopsis***. **A**. Expression of *TCL2 *and *TCL1 *in different tissues of Arabidopsis. **B**. Expression of *TCL2 *in pedicels. Tissues from different parts of wild-type *Arabidopsis *were collected, and RNA was isolated, then RT-PCR was used to check the expression of *TCL2 *and *TCL1*. The expression of *ACTIN2 *(*ACT2) *was used as a control.

To characterize the function of TCL2, we first examined the expression of *TCL2 *across various tissues and organs. We also compared the expression pattern of *TCL2 *with that of *TCL1 *because TCL2 is more closely related to TCL1 than other single-repeat MYBs at the amino acid level. Various tissues and organs of wild-type plants were harvested, and RT-PCR was used to examine the expression of *TCL2 *and *TCL1*. We found that *TCL2 *is expressed in all tissues/organs tested except young siliques, where *TCL1 *is highly expressed (Figure 2). *TCL2 *is also highly expressed in cotyledons, organs that normally do not produce any trichomes and where the transcript of *TCL1 *was at undetectable level (Figure 2). To get more details about when, where and in response to what stimulus *TCL2 *is expressed, we then searched public available gene expression database genevestigator (http://www.genevestigator.com/gv) using At2g30424 as gene ID, however, results showed that there is no probeset could be found for the gene ID provided, possibly because *TCL2 *is previously unidentified gene, and no probes for this gene have been printed on microarrays used for all those experiments that provided data for the database.

**Figure 3:**
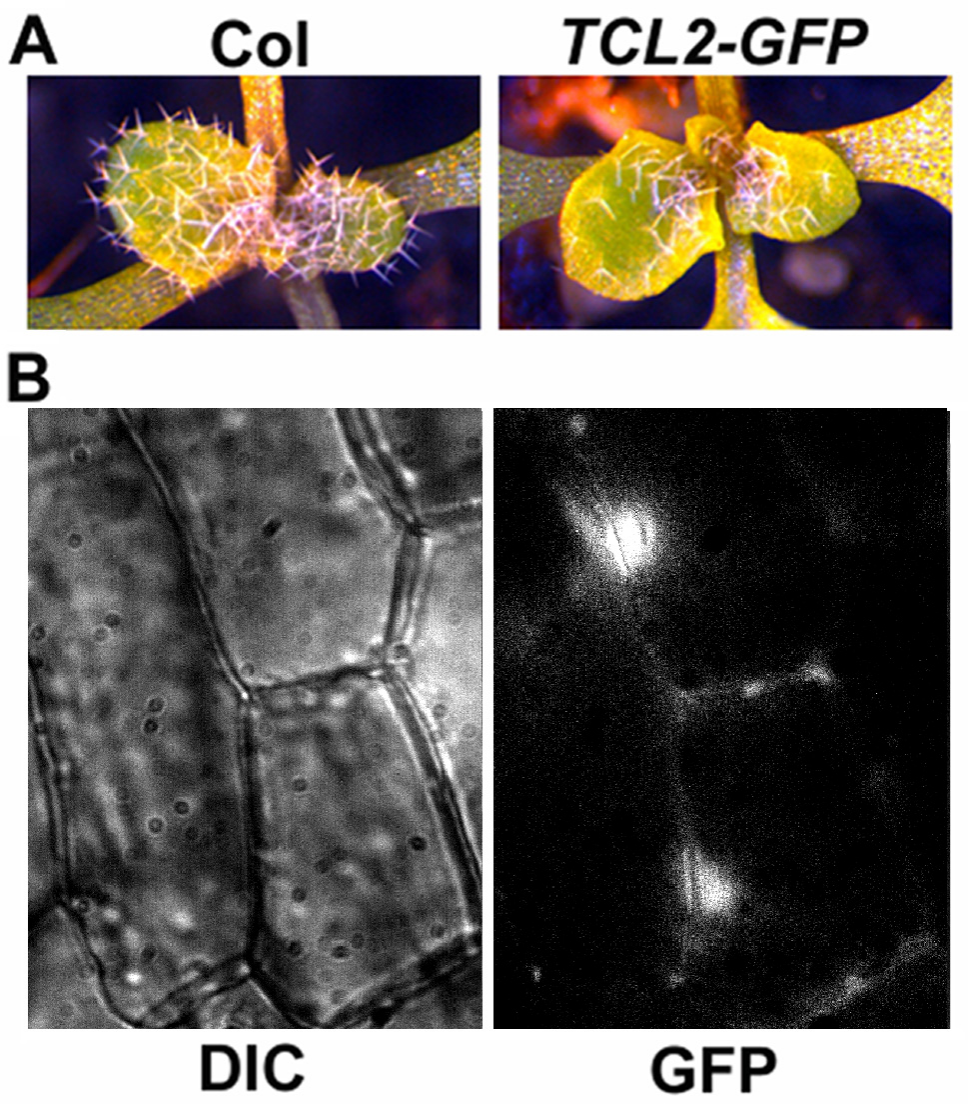
**Subcellular localization of TCL2**. **A**. Expression of TCL2-GFP in Arabidopsis resulted in reduced number of trichomes. Left panel: wild-type, right panel: *PTCL2:TCL2-GFP *transgenic plant. **B**. TCL2-GFP fluorescence in the epidermal cells of the hypocotyls in a 4-day-old transgenic plant expressing *TCL2-GFP *under the control of *TCL2 *promoter. Left panel: DIC channel, right panel: GFP channel.

**Figure 4:**
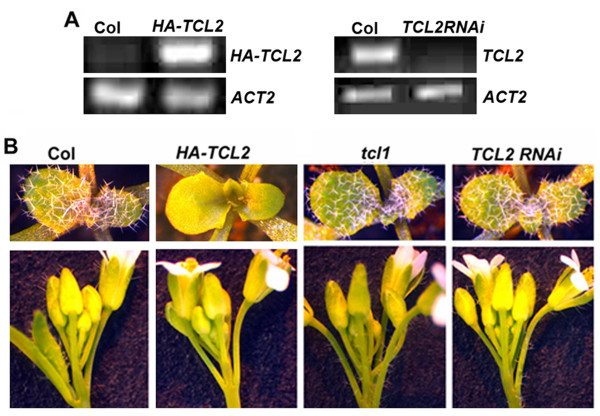
**TCL2 negatively regulates trichome formation on the inflorescence stems and pedicels**. **A**. Expression of *TCL2 *in *35S:HA-TCL2 *and *35S:TCL2RNAi *transgenic plants. RNA was isolated form 10-day-old seedlings and RT-PCR was used to check the expression of *TCL2*. HA specific and gene specific primers were used to detect *HA-TCL2*, and gene specific primers were used to detect *TCL2*. The expression of *ACTIN2 *(*ACT2) *was used as a control. **B**. Glabrous phenotype in plant overexpressing TCL2 and ectopic trichome formation in *TCL2 *knockdown plants. From left to right: Rosette leaves (upper) and inflorescences (Lower) of Col, *HA-TCL2*, *tcl1 *and *TCL2RNAi *plants.

To examine TCL2's subcellular localization, we generated transgenic plants expressing TCL2-GFP fusion protein under the control of *TCL2*'s own promoter, a genomic DNA fragment that covers the region -1502 to +1 of the start codon of *TCL2*. The transgenic plant produces reduced number of trichomes (Figure 3A), a phenotype similar to that of *PTCL1:TCL1-GFP *transgenic plant [13],indicating that the reduction in trichome number is due to an increase on the copy number of the *TCL2 *gene, as overexpression of *TCL2 *showed a greatly reduced trichome phenotype (Figure 4). These results also indicate that both the *TCL2 *promoter and TCL2-GFP fusion protein are likely functional. By using the transgenic plants obtained, we showed that TCL2-GFP proteins are mainly observed in the nucleus of epidermal cells (Figure 3B).

### TCL2 is a negative regulator of trichome formation

To analyze the function of *TCL2 *in trichome formation, we generated transgenic plants overexpressing HA tagged TCL2 under the control of *35S *promoter (*35S:HA-TCL2*). Transcript levels of *TCL2 *were examined by RT-PCR using *HA*-specific and *TCL2 *specific primers (Figure 4A). As observed previously for all other single-repeat R3 MYB transcription factors, transgenic plants overexpressing *TCL2 *resulted in glabrous phenotypes, with no trichome formation on rosette leaves, inflorescence stems, cauline leaves or floral organs (Figure 4B).

To further analyze the function of TCL2 in trichome formation, we sought loss-of-function alleles of *TCL2*. By searching the SALK T-DNA Express Database, we found there are four T-DNA insertion mutants that are related to *TCL2*. However, in all those four lines, the T-DNA was inserted in the promoter regions, and expression of *TCL2 *is largely unaffected as examined by RT-PCR (data not shown). As a result, all the mutants have wild-type trichome phenotypes.

Therefore, we took RNAi approach to knock down the expression of *TCL2*. Since *TCL2*'s ORF fragment is relative small, only 303 bp in length, we used the full-length ORF to create the RNAi construct. We selected three independent transgenic lines displaying ectopic trichome formation on inflorescence stems and pedicels (Figure 4B), similar to that of *tcl1 *mutant [13]. RT-PCR results showed that expression level of *TCL2 *in the transgenic lines was dramatically reduced (Figure 4A), while expression of other single-repeat MYB genes was largely unaffected (see Additional file 1), indicating that the phenotype observed is caused by down-expression of *TCL2*.

**Figure 5:**
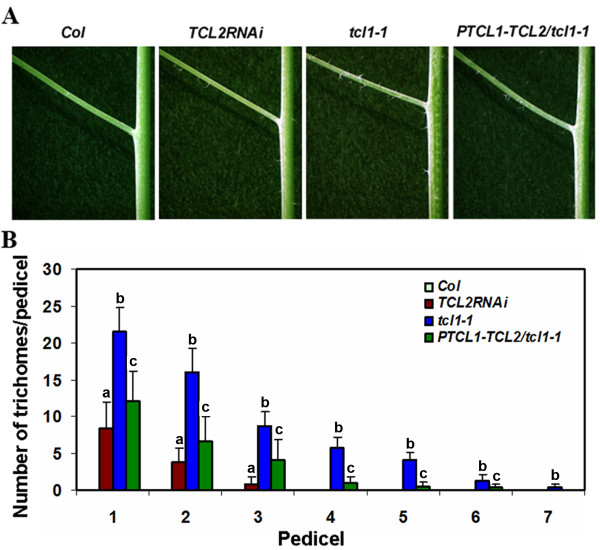
**TCL2 has overlapping function with TCL1 to regulate trichome formation on the inflorescence stems and pedicels**. **A**. Trichome formation on the first pedicel and inflorescence internodes in Col, *TCL2RNAi*, *tcl1-1 *and *PTCL1-TCL2*/*tcl1-1 *plants. **B**. Trichome formation on pedicels of Col, *TCL2RNAi*, *tcl1-1 *and *PTCL1-TCL2*/*tcl1-1 *plants. Data represent mean ± SD of at least 10 plants for each genotype. a: significantly different from WT (*P *< 0.01), b: significantly different from TCL2RNAi mutant (*P *< 0.01), c: significantly different from *tcl1 *mutant (*P *< 0.05).

When we quantified the number of trichomes on internodes and pedicels, we found that compared with *tcl1 *mutant, there are fewer internodes and pedicels bearing ectopic trichomes in *TCL2RNAi *mutant (Figure 5).

### *TCL2 *partially rescued *tcl1 *mutant trichome phenotypes

Previously, we identified *TCL1 *as a major single-repeat MYB transcription factor that negatively regulates trichome formation on the inflorescences and pedicels [13]. The *tcl1 *mutant confers ectopic trichome formation on the inflorescence stems and pedicels. Here we showed that transgenic plants with reduced expression of *TCL2 *have similar phenotype as that of *tcl1 *(Figure 4B, Figure 5). Loss-of-function mutations in any other single-repeat MYBs including *TRY*, *CPC*, *ETC1*, *ETC2*, and *ETC3 *do not result in similar inflorescence and pedicel trichome phenotypes as shown in *tcl1 *mutant or *TCL2RNAi *lines. Therefore, we wanted to further examine the relationship between *TCL2 *and *TCL1 *in trichome formation on the inflorescence stems and pedicels. Double mutant was generated between *tcl1 *and *TCL2RNAi *mutants, however, the double mutant was indistinguishable from *tcl1 *single mutant (data not shown), possibly because higher order redundancy function among single repeat genes [19]. Therefore we turned to test if *TCL2 *could rescue *tcl1 *mutant phenotype. Transgenic plants were generated to express *TCL2 *under the control of *TCL1 *promoter in a *tcl1 *background (*P*_*TCL1*_*:TCL2/tcl1*). Our previous results have showed that expression of TCL1-GFP fusion protein under the control of *TCL1 *promoter fully rescued *tcl1 *trichome phenotype [13], indicating that the *TCL1 *promoter used is fully functionally. As shown in Figure 5, expression of TCL2 protein under the *TCL1 *promoter only partially rescued *tcl1 *mutant phenotype, indicating that TCL2 is functionally similar but not identical to TCL1 in controlling trichome patterning on the inflorescence stems and pedicels.

### TCL2 interacts with GL3

It has been proposed that single-repeat MYB transcription factors control trichome formation by competing with GL1 for binding GL3, thus blocking the formation of TTG1-GL3-GL1 activator complex. We have previously demonstrated that TRY, CPC, TCL1, ETC1, ETC2, and ETC3, interact with GL3 using a protoplast transfection system [19]. To test if TCL2 controls trichome formation using a similar mechanism, we tested if TCL2 interacts with GL3 in plant cells.

**Figure 6:**
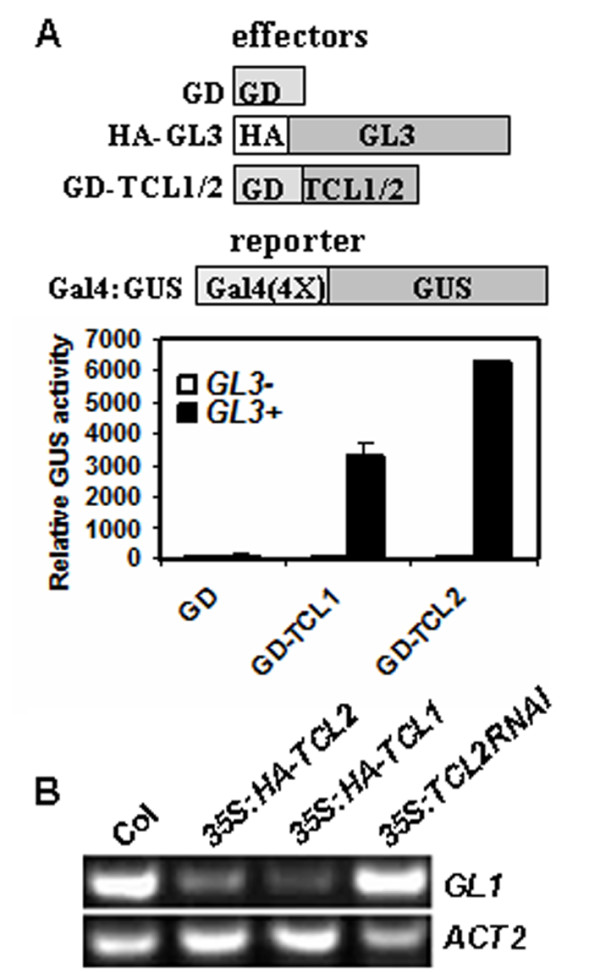
**TCL2 interacts with GL3 in plant cells and affects *GL1 *expression**. **A**. TCL2 interacts with GL3 in plant cells. Reporter gene and effector genes were cotransfected into protoplasts derived from Arabidopsis rosette leaves. GUS activity was measured after 20-22 h incubation of the protoplasts in darkness. Data represent the mean ± SD of three replicates. Effectors and reporter constructs were diagrammed at the top of the figure. **B**. Regulation of *GL1 *by TCL2. RNA was isolated from 10-day-old seedlings of Col, *35S:HA-TCL2*, *35S:HA-TCL1 *and *35S:TCL2RNAi*, and RT-PCR was used to examine the expression of *GL1*. Expression of *ACT2 *was used as a control.

A protoplast transient expression system was used to test the interaction between TCL2 and GL3. A *Gal4-GUS *reporter, together with the effectors *GL3 *and a Gal4 DNA binding domain (GD) fused TCL2 (*GD-TCL2*) were co-transfected into *Arabidopsis *protoplasts. *GD *alone and *GD-TCL1 *were used as negative and positive control, respectively. As shown in Figure 6A, with or without GL3, GD alone could not activate the expression of the reporter gene. In the absence of GL3, neither GD-TCL1 nor GD-TCL2 activated the expression of the reporter gene presumably due to the fact that single-repeat R3 transcription factors do not have activation domains. However, in the presence of GL3, both GD-TCL2 and GD-TCL1 activated the reporter gene, indicating that TCL2 interacts with GL3 in plant cells. It should be noted that GL3 can function as a transcriptional activator, however, alone it cannot be recruited to the *Gal4 *DNA binding elements in the reporter gene [20]. It was also observed that the expression of the reporter gene activated by GD-TCL2 in the presence of GL3 is much higher than that of GD-TCL1 (Figure 6A), implying that the binding affinity between TCL2 and GL3 may be higher than that between TCL1 and GL3.

### TCL2 suppresses the expression of *GL1*

In addition to competing with GL1 for binding GL3, we also reported previously that TCL1 can directly suppress the expression of *GL1 *[13]. Because TCL2 is more closely related to TCL1 than any other single-repeat MYBs at the amino acid level (Figure 1) and *TCL2RNAi *lines displayed similar inflorescence and pedicels trichome phenotypes as *tcl1 *mutants, we then tested if TCL2 also affects the expression of *GL1*. As shown in Figure 6B, expression level of *GL1 *is dramatically reduced in *35S:HA-TCL2 *seedlings, similar to that in *35S:HA-TCL1 *seedlings, suggesting that TCL2 can also suppress the expression of *GL1*.

### Neither GL1-GL3 activator complex nor SPLs alone activates *TCL2*

**Figure 7:**
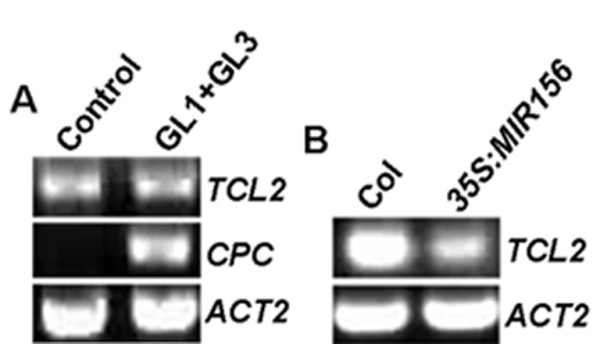
**Regulation of *TCL2***. **A**. Expression of *TCL2 *is not regulated by GL1-GL3 activator complex. GL1 and GL3 were cotransfected into protoplasts derived from *Arabidopsis *rosette leaves, RNA was isolated from transfected protoplasts after 20-22 h incubation in darkness, and RT-PCR was used to examine the expression of *TCL2 *and *CPC*. Expression of *ACT2 *was used as a control. **B**. Expression of *TCL2 *is down-regulated in *35S:MIR156 *plants. RNA was isolated from 10-day-old seedlings of Col and *35S:MIR156*, and RT-PCR was used to examine the expression of *TCL2*. Expression of *ACT2 *was used as a control.

Available evidence suggested that TTG1-GL3-GL1 activator complex activates both *GL2 *and some single-repeat MYB genes. Our previous reporter showed that GL1 and GL3 are required and sufficient to activate *GL2 *and a subset of single-repeat MYB genes including *TRY*, *CPC*, *ETC1 *and *ETC3*, but not *ETC2 *and *TCL1*[19,20]. To test if *TCL2 *is regulated by GL1-GL3 complex, GL1 and GL3 were co-transfected into protoplasts and RT-PCR was used to examine the expression of *TCL2*. Expression of *CPC *was used as a positive control. As expected, *CPC *was strongly induced by GL1 + GL3. However, such activation was not observed for *TCL2 *(Figure 7A).

We wanted to further investigate the molecular mechanism controlling the activation of *TCL2 *transcription. Recently, it has been showed that *TCL1 *is directly activated by *MIR156 *directed SPLs [21]. In order to explore the possibility that expression of *TCL2 *may also be controlled by *MIR156 *directed SPLs, we tested the expression of *TCL2 *in *35S:MIR156 *plant. Indeed, we found that the expression level of *TCL2 *in *35S:MIR156 *plant was dramatically reduced (Figure 7B), similar to that of *TCL1 *[21]. To further test if SPLs can directly active *TCL2*, we cloned five SPL genes including *SPL3*, *SPL9*, *SPL10*, *SPL13 *and *SPL15*, transiently expressed them in protoplasts, and then used RT-PCR to examine the expression of *TCL2*. The five SPLs were chosen because all of them but SPL15 have been shown to regulate trichome formation [21], while SPL15 is closely related to SPL9 [24]. To our surprise, no significant changes were observed (data not shown). Since over-expression of SPLs in plants has already been shown to be able to induce the expression of *TCL1*, and SPL9 has been shown to directly bind to the promoter region of *TCL1 *[21], we test if the expression of *TCL1 *in protoplasts transfected with SPLs is affected. However, we did not see any significant changes (data not shown). These results suggested that SPLs alone may not be sufficient to activate the expression of *TCL1*. To explore this possibility further, all five SPLs cloned were fused with GD and co-transfected with *Gal4:GUS *reporter gene into protoplasts, then GUS activities were assays. Indeed, none of the five SPLs tested could activate the reporter gene (data not shown). These results indicated that although SPLs can bind directly to the promoter region of *TCL1*, they might require co-activators to regulate the expression of their target genes, including *TCL1*, *TRY *and possibly, *TCL2*.

## Discussion

### TCL2 is a negative regulator of trichome formation

In this study we analyzed the function of *TCL2*, a previously uncharacterized single-repeat R3 MYB transcription factor in trichome formation in *Arabidopsis*. We showed that *TCL2*, like *TCL1*, *TRY*, *CPC*, *ETC1*, *ETC2 *and *ETC3*, act as a negative regulator for trichome formation. Overexpression of *TCL2 *under the control of the *CaMV 35S *promoter resulted in a glabrous phenotype (Figure 4). Knockdown of *TCL2 *via RNAi (*TCL2RNAi*) resulted in ectopic trichome formation on the inflorescence stems and pedicels (Figure 4), while the number and patterning of trichomes on leaves were largely unaffected, a phenotype similar to that of *tcl1 *[13]. These results suggest that TCL2 negatively regulates trichome formation and that TCL2 may have overlapping function with TCL1 in controlling trichome formation on the inflorescence stems and pedicels.

We also showed that expression of *TCL2 *under the control of the *TCL1 *promoter in a *tcl1 *mutant background (*P*_*TCL1*_*:HA-TCL2/tcl1*) could only partially rescue the trichome phenotype of the *tcl1 *mutant. Trichomes were still present on the inflorescence stems and pedicels of *P*_*TCL1*_*:TCL2/tcl1 *plants, although the frequency was significantly reduced (Figure 5). On the other hand, expression of *TCL1 *under the control of the *TCL1 *promoter in *tcl1 *mutant background fully rescued the trichome phenotype of the *tcl1 *mutant [13]. These results implied that TCL2 and TCL1 may function redundantly to control trichome development on inflorescence stems and pedicels but their function may not fully interchangeable.

Although transcript of *TCL2 *is detectable in roots (Figure 2), all the mutants tested including *TCL2 *overexpressor, *TCL2RNAi *and *P*_*TCL1*_*:TCL2/tcl1 *are largely indistinguishable from wild type in term of root hair formation (data not shown). However, we could not rule out the possibility that TCL2 may play a role in regulating root hair formation, a higher order combination of mutations in single repeat MYBs may help clarify the exact role of TCL2 in root formation.

### How does TCL2 regulate trichome formation

Previous research has pointed out that trichome formation is regulated by an activator/inhibitor feedback system [7,10,19,25]. TTG1, GL1, and GL3 or EGL3 form an activator complex (TTG1-GL3/EGL3-GL1) that initiates trichome development by activating *GL2*. The same activator complex also activates some single-repeat R3 MYB transcription factor genes, whose products, in turn, compete with GL1 for binding GL3/EGL3, thus inhibiting the formation of the activator complex. Our previous study suggested that TCL1 could also directly suppress the expression of *GL1*, providing another negative feedback loop on the regulation of trichome formation [13]. By using a protoplast transfection system, we showed that TCL2 interacts with GL3 (Figure 6A), suggesting that TCL2 could block the formation of the activator complex required for trichome formation (see Additional file 2). RT-PCR results showed that expression of *GL1 *is dramatically reduced in the plants overexpressing *HA-TCL2*, indicating the TCL2 can also suppress the expression of *GL1*. These results suggest that TCL2 negatively regulate trichome formation in a manner similar to that of TCL1 (see Additional file 2). However, we could not rule out the possibility that TCL2 may also regulate trichome formation through other mechanisms, since our recent results showed that single-repeat MYBs may regulate trichome formation independent of GL2 [26].

### Regulation of *TCL2*

Available evidence supports that TTG1-GL3/EGL3-GL1 activator complex plays an important role in regulating trichome formation. Recently, several phytohormones, such as jasmonic acid (JA), gibberellins (GAs) and cytokinins are found to be involved in the promotion of trichome formation, and this promotion is mediated, at least in part, by transcriptional regulation of the components of TTG1-GL3/EGL3-GL1 complex [27-30], providing further support for the critical role of the activator complex in the regulation of trichome formation. In addition to regulating the expression of *GL2 *to promote trichome formation, the activator complex also activates some single-repeat MYB genes to provide a negative feedback loop. Available evidence suggested that the transcription of *CPC*, *TRY*, *ETC1*, and *ETC3*, but not *TCL1 *and *ETC2*, is regulated by the proposed activator complex [19,31], implying that other pathways may also be involved in the regulation of single-repeat MYB genes. Recently, it has been showed that *MIR156*-regulated SPLs, which promote phase transition, directly activate *TCL1 *and *TRY *expression through binding to their promoters and that this activation is independent of *GL1*[21].

Using protoplast transient expression system, we found that the expression of *TCL2 *is not regulated by TTG1-GL3/EGL3-GL1 complex (Figure 7A). Reduced expression of *TCL2 *in *35S:MIR156 *transgenic plants suggested that *MIR156*-targeted SPLs may also regulate the expression of *TCL2 *(Figure 7B). However, when expressed in protoplasts, none of the five SPLs tested could induce the expression of neither *TCL2 *nor *TCL1*. Further test suggested that SPLs alone could not activate reporter gene expression when recruited to the promoter region of the reporter gene by a fused DNA binding domain. These results indicated that there might be other co-activator working together with SPLs to regulate the expression of *TCL1*, and possible *TCL2 *(see Additional file 2). It is also possible that unidentified targets of *MIR156 *may involve in the regulation of single-repeat MYB genes.

## Conclusions

In this report, we identified TCL2 as a new member of single-repeat MYB transcription factor family, and provided evidence to show that TCL2 is a negative regulator of trichome development. We showed that TCL2 may have overlapping function with TCL1 to regulate trichome formation, but its function is not fully interchangeable with TCL1. Furthermore, we showed that TCL2 interact with GL3 in plant cells, its expression is not regulated by GL3/GL1 activator complex, and that *MIR156 *is involved in the regulation of *TCL2*. Finally, we found that although SPLs can bind directly to the promoter region of *TCL1*, they likely require co-activators to regulate the expression of their target genes, including *TCL1*, *TRY*, and possibly, *TCL2*.

## Methods

### Plant materials and growth conditions

The *tcl1 *mutant, *35S:MIR156 *and *35S:HA-TCL1 *transgenic plant are in the Columbia-0 (Col-0) background [13,21]. T-DNA insertion lines related to *TCL2 *were obtained from SALK Institute.

Seedlings used for RT-PCR analysis were grown on 1/2 Murashige & Skoog (MS) medium with vitamins (plantmedia) and 1% (w/v) sucrose. Seedlings used for phenotypic analysis were obtained either by plating seeds on 1/2 MS medium or by directly sowing seeds into soil. Plants were grown at 22 C with 14/10 hour photoperiod at approximately 120 μmol m^-2 ^s^-1^.

### Constructs

*35S:GD-TCL1*, *35S:HA-GL3 *have been described previously [13]. To generate HA or GD tagged constructs for *TCL2*, *SPL3*, *9*, *10*, *13 *and *15*, the full-length open-reading frames (ORF) of corresponding genes were amplified by RT-PCR using RNA from 10-day-old light-grown Arabidopsis seedlings, then the PCR products were cloned in frame with an N-terminal HA or GD tag into the *pUC19 *vector under the control of the double *35S *enhancer promoter of CaMV. The *P*_*TCL2*_*:HA-TCL2-GFP *was cloned by fusing *TCL2 *in frame with GFP and then subcloned into the *pUG19 *vector under the control of the *TCL2's *own promoter (a fragment that covers the region -1502 to +1 of the start codon of *TCL2*). For plant transformation, corresponding constructs in *pUG19 *vector were digested with *EcoRI*, then subcloned into the binary vector *pPZP211 *or *pPZP221 *[32].

To generate *TCL2RNAi *construct. The *TCL2 *fragment was amplified by RT-PCR using forward primer 5' -CAACTCGAGGATAACACCAACCGTCT-3' and reverse primer 5'-CAAGAATTCAGGAGGAGAAATAGAGA-3', which contain an *XhoI *and an *EcoRI *enzyme cutting site at its 5'-end respectively. The PCR product was digested by *XhoI *and *EcoRI *and cloned into vector *pHANNIBAL*. The vector contained the *CaMV 35S *promoter and the *nos *terminator in sense orientation. The resulting plasmid was named as *pHANNIBAL-TCL2S*. The anti-sense fragment was amplified by RT-PCR using forward primer 5'-CAATCTAGAGATAACACCAACCGTCT-3' and reverse primer 5'-CAAAAGCTTAGGAGGAGAAATAGAGA-3', which contain an *XbaI *site and a *HindIII *enzyme cutting site at its 5'-end respectively. The PCR product was digested by *XbaI *and *HindIII *and cloned into *pHANNIBAL-TCL2S *plasmid, resulting *pHANNIBAL-TCL2RNAi *construct. The *TCL2RNAi *cassette was then removed from *pHANNIBAL-TCL2RNAi *by digestion with *NotI*, and subcloned into binary vector *pART27 *for plant transformation.

### Phylogenetic analysis

Full length protein sequences of TRY, CPC, TCL1, TCL2, ETC1, ETC2 and ETC3/CPL3 were used for phylogenetic analysis. Multiple sequence alignment and dendrogram with branch length were generated using CLUSTAL W Multiple Sequence Alignment Program with default settings (http://www.genome.jp/tools-bin/clustalw).

### Plant transformation and selection of transgenetic plants

About five-week-old plants with several mature flowers on the main inflorescence were used to transform with various constructs via *Agrobacterium tumefaciens *GV3101 by using the floral dip method [33]. Phenotypes of transgenic plants were examined in the T1 generation, and confirmed in T2 up to T4 generations. Up- or down-expression of corresponding genes in related lines was confirmed by RT-PCR. For each construct, at least three independent transgenic lines with similar phenotypes were obtained, and results from a representative line were presented.

### Plasmid DNA isolation, protoplast transfection and GUS activity assay

Plasmid DNA was prepared using the Qiagen EndoFree Plasmid Maxi Kit (http://www.qiagen.com). Protoplast isolation, transfection and the GUS activity assay were performed as described previously [34-36]. Briefly, protoplasts were isolated from rosette leaves of ~4-week-old plants. Effector and reporter plasmids were co-transfected into protoplasts and incubated under darkness for 20-22 h. GUS activities were measured using a BioTEK Synergy™ HT microplate reader (http://www.biotek.com). Expression of *35S:luciferase *(Luc) was used to normalize the expression of the GUS reporter. Luciferase activities were measured using a microplate luminometer (http://www.turnerdesigns.com) together with the Promega Steady-Glo luciferase assay system (http://www.promega.com/).

### Microscopy

Trichomes were analyzed and photographed as described previously [13,19].

Four-day-old seedlings expressing *TCL2-GFP *under the control of the *TCL2 *promoter were used to examine the expression and localization of TCL2-GFP, by using a Leica DM-6000B upright fluorescent microscope with phase and differential interference contrast (DIC), photos were taken using a Leica FW4000 digital camera connected to the microscope (http://www.leica-microsystems.com).

### RNA isolation and RT-PCR

Total RNA was isolated from different parts of plant, seedlings or transfected protoplasts using the RNeasy Plant Mini Kit (QIAGEN, Mississauga, Ontario, Canada). cDNA was synthesized using 2 μg total RNA by Oligo(dT)-primed reverse transcription using OMNISCRIPT RT Kit (QIAGEN). Some of the primers used for cloning or examining the expression of corresponding genes have been described previously [13,19], and others are as follows. *TCL2*-specific primers (5'-CATATGGATAACACCAACCGTCT-3' and 5'-GAGCTCTTAAGGAGGAGAAATAG-3') were used to amplify the full-length ORF of TCL2. *HA*-specific (5'-TACCCTTACGATGTTCCTGATTAC-3') and TCL2-specific (5'-GAGCTCTTAAGGAGGAGAAATAG-3') primers were used to check the expression of *HA-TCL2*. *TCL2RNAi*-specific primers (5'-GTCCTCTTTCACTTTCAAATACCAATG-3' and 5'-CTTAAAGCTTTTATCTTGTCC-3') were used to check the expression of *TCL2 *in *TCL2RNAi *transgenic plants.

## Competing interests

The authors declare that they have no competing interests.

## Authors' contributions

LG carried out most of the experiments. SW designed the experiments and carried out some of the experiments. LG and SW drafted the manuscript. KX and J-GC conceived of the study, participated in its design and helped to edit the manuscript. All authors read and approved the final manuscript.

## Supplementary Material

Additional file 1 **Adobe Photoshop, Expression of single repeat MYB genes in *35S:TCL2RNAi *transgenic plants**. RNA was isolated from 10-day-old seedlings of wild type and two independent *35S:TCL2RNAi *transgenic lines, and RT-PCR was used to check the expression of single repeat MYB genes. Note that transcript of *ETC1 *was not included in the figure because we were unable to detect the expression of *ETC1 *in seedlings. The expression of ACTIN2 (*ACT2) *was used as a control. Click here for file

Additional file 2 **Adobe Photoshop, Model of the transcription factor network that controls trichome formation in *Arabidopsis***. TTG1, GL3/EGL3 and GL1 form an activator complex, to regulate the transcription of *GL2*, and some of the single repeat MYB genes. Single MYB transcription factors, in turn, compete with GL1 for binding of GL3/EGL3, thus limiting the formation of the TTG1-GL3-GL1 activator complex. As shown in this study, TCL2 can intact with GL3, it can also suppress the expression of *GL1*, thereby limiting the transcriptional activity of the TTG1-GL3-GL1 activator complex. Like that of *TCL1*, transcription of *TCL2 *is regulated by SPLs, however, unknown co-activator may required for the transcriptional activation. Question marks indicate unknown regulators or unclear processes. Click here for file
