# Supplementary figures and images for: Functional characterization of TRICHOMELESS2, a new single-repeat R3 MYB transcription factor in the regulation of trichome patterning in Arabidopsis
Source: BMC Plant Biol. 2011 Dec 15;11:176. doi: 10.1186/1471-2229-11-176 (PMC3264604; doi:10.1186/1471-2229-11-176)

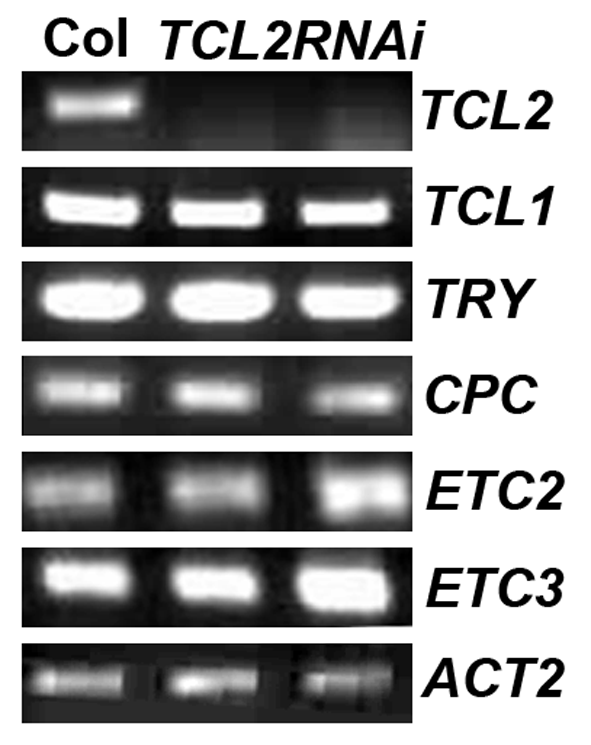

Supplement: Additional file 1 — Adobe Photoshop, Expression of single repeat MYB genes in 35S:TCL2RNAi transgenic plants. RNA was isolated from 10-day-old seedlings of wild type and two independent 35S:TCL2RNAi transgenic lines, and RT-PCR was used to check the expression of single repeat MYB genes. Note that transcript of ETC1 was not included in the figure because we were unable to detect the expression of ETC1 in seedlings. The expression of ACTIN2 (ACT2) was used as a control. [file 1471-2229-11-176-S1.TIFF]

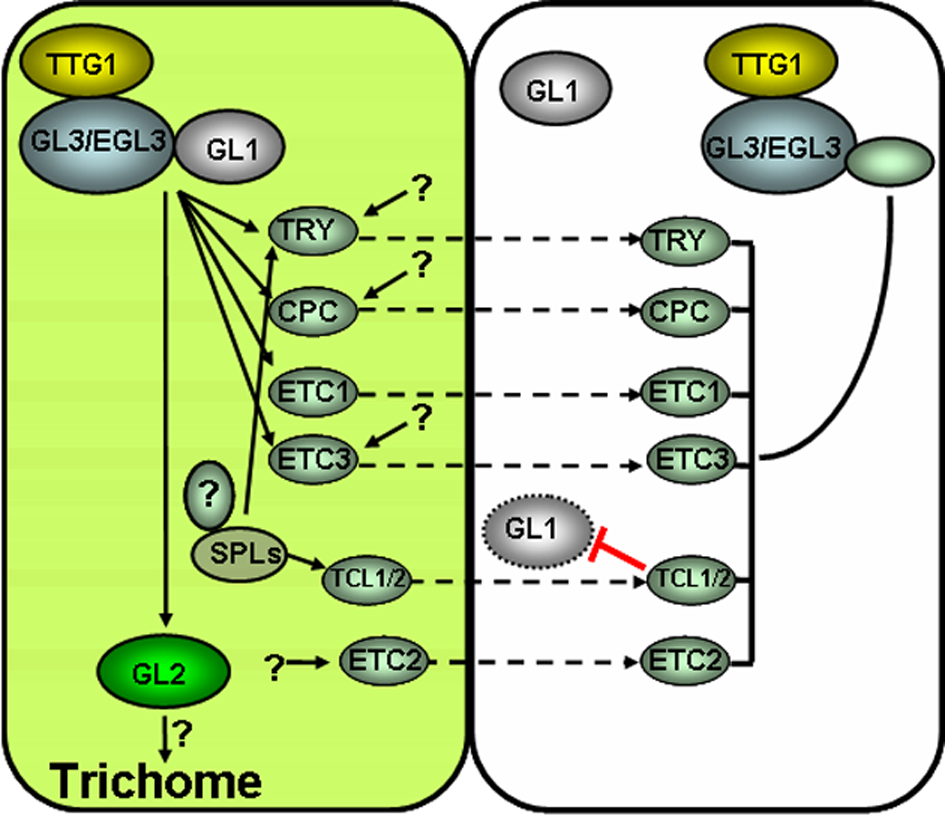

Supplement: Additional file 2 — Adobe Photoshop, Model of the transcription factor network that controls trichome formation in Arabidopsis. TTG1, GL3/EGL3 and GL1 form an activator complex, to regulate the transcription of GL2, and some of the single repeat MYB genes. Single MYB transcription factors, in turn, compete with GL1 for binding of GL3/EGL3, thus limiting the formation of the TTG1-GL3-GL1 activator complex. As shown in this study, TCL2 can intact with GL3, it can also suppress the expression of GL1, thereby limiting the transcriptional activity of the TTG1-GL3-GL1 activator complex. Like that of TCL1, transcription of TCL2 is regulated by SPLs, however, unknown co-activator may required for the transcriptional activation. Question marks indicate unknown regulators or unclear processes. [file 1471-2229-11-176-S2.TIFF]
